# Financial, institutional, environmental, technical, and social (FIETS) aspects of water, sanitation, and hygiene conditions in indigenous - rural Indonesia

**DOI:** 10.1186/s12889-021-11800-x

**Published:** 2021-09-22

**Authors:** D. Daniel, Dennis Djohan, Ilias Machairas, Saket Pande, Arifin Arifin, Trimo Pamudji Al Djono, Luuk Rietveld

**Affiliations:** 1 Department of Water Management, Faculty of Civil Engineering and Geosciences, Delft University of Technology, Delft, the Netherlands; 2 Department of Health Behaviour, Environment, and Social Medicine, Faculty of Medicine, Public Health and Nursing, Universitas Gadjah Mada, Yogyakarta, Indonesia; 3 Department of Environmental Engineering, Faculty of Civil Engineering and Geosciences, Delft University of Technology, Delft, the Netherlands; 4 Department of Groundwater Engineering, Faculty of Earth Sciences and Technology, Bandung Institute of Technology, Bandung, Indonesia; 5 Department of Environmental Engineering, Sekolah Tinggi Teknologi Sapta Taruna, Jakarta, Indonesia

**Keywords:** Water supply, Sanitation, Qualitative analysis, Sustainability, Culture, FIETS

## Abstract

**Background:**

There is increasing recognition of the complexity underlying WASH conditions in developing countries. This article explores the complexity by assessing the vulnerability of a specific area to poor WASH conditions using a qualitative approach.

**Methods:**

We present our findings for the district of East Sumba in Indonesia. This area is known as one of the poorest regions in Indonesia with inadequate WASH services, indigenous belief that hinder the practice of WASH-related behaviours, and has a high rate of children malnutrition. All the factors that contribute to poor WASH conditions were discussed through the lens of the Financial, Institutional, Environmental, Technological, and Social (FIETS) framework. We then summarised the factors and visualized the “system” using a mind map which shows how factors are interconnected and helps to find the root causes of poor WASH conditions.

**Results:**

There are three main challenges that inhibit the improvement of WASH conditions in this area: inadequate institutional capacity, water scarcity, and poor socio-economic conditions. We found that a village leader is the most important actor who influences the sustainability of WASH services in this area and healthcare workers are influential WASH promoters. This study also shows how culture shapes people’s daily lives and institution performance, and influences the current WASH conditions in East Sumba. The mind map shows there is an overlap and interconnection between FIEST aspects and WASH conditions in the study area.

**Conclusion:**

WASH conditions are influenced by many factors and are often interconnected with each other. Understanding this complexity is necessary to improve WASH conditions and sustain adequate WASH services in developing countries. Finally, WASH interventions have to be considerate of the prevailing cultural practices and should involve multidisciplinary stakeholders.

**Supplementary Information:**

The online version contains supplementary material available at 10.1186/s12889-021-11800-x.

## Introduction

Water supply, sanitation, and hygiene (WASH) are focal points in the Sustainable Development Goal (SDG) 6, which aims to “ensure availability and sustainable management of water and sanitation for all” [1]. The latest WHO/UNICEF Joint Monitoring Programme (JMP) report showed the progress made in the provision of WASH services in 2017: 71% of the global population had safely managed drinking water services, 45% safely managed sanitation services, and 60% of the global population had basic handwashing facilities with soap and water availability at home [2]. This indicates that billions of people, especially in low-middle income countries (LIMCs), still lack adequate WASH services. Moreover, lack of adequate WASH services contributes to disease, e.g., diarrhea and stunting, and death cases, especially among children under five in LMICs [3].

Dreilbelbis et al. [4] argue that previous WASH-related behavioral studies were more focused on factors related to the individual level and little attention was given to other factors, such as technological, environmental, and institutional aspects, which contribute significantly to WASH behaviour and associated conditions. For example, water scarcity, i.e., an environmental aspect, can influence WASH-related behaviour [5, 6]. Another example is that the institutional quality influences the sustainability of rural water supply programs [7], which then can influence people’s behaviour.

**Fig. 1:**
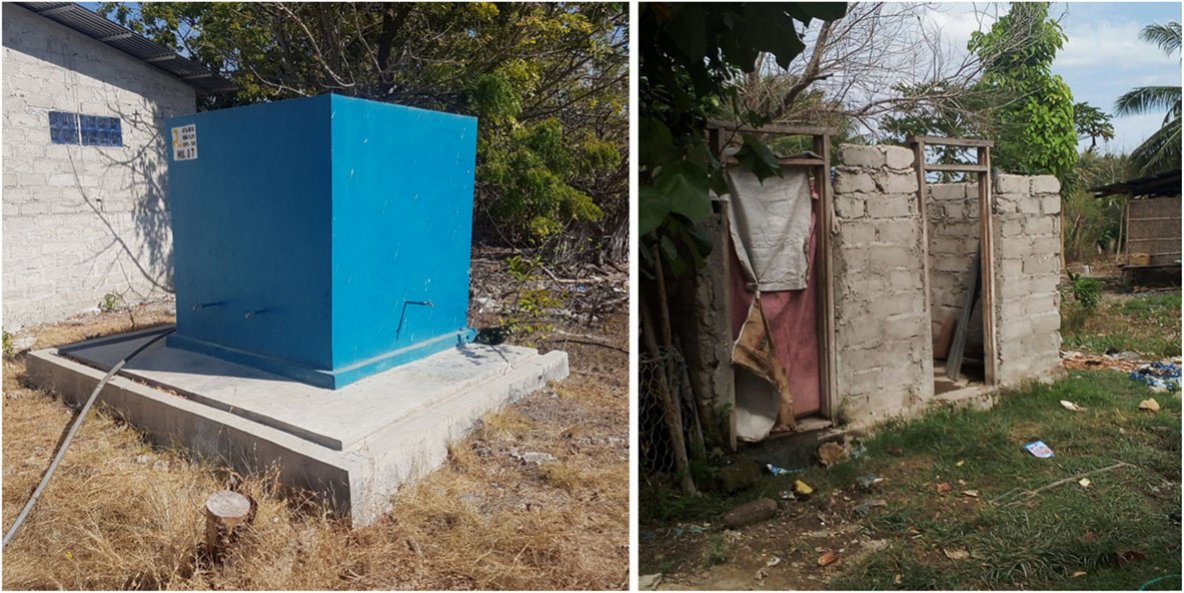
Examples of unsustainable WASH services in East Sumba. Left: A not-functional water tank; Right: An unfinished toilet construction. Pictures were taken by the first author

Indonesia is still struggling to provide 100% access to safely managed drinking water and sanitation services to its citizens. Only 89% of Indonesia’s population benefited from “basic” drinking water services, despite significant progress in reducing open defecation from 33% in 2000 to 10% in 2017 [2]. The prevalence of child stunting in Indonesia remains high, 30.8% in 2018 [8]. Moreover, the continuation of WASH services over a long period is still a challenge in many places in Indonesia (Fig. 1) [9], which highlights the need to analyse it further.

To the best of our knowledge, there is no WASH study that qualitatively evaluates the *financial*, *institutional*, *environmental*, *technological*, and *social* (FIETS) aspects that influence WASH conditions. Thus, the objective of this paper is to identify the factors that contribute to the sustainability of WASH services by considering FIETS aspects. This qualitative approach can help reveal how and why a phenomenon exists, e.g., WASH conditions in a specific area [10].

East Sumba, which is one of the districts of East Nusa Tenggara province, was used as the study area. This area is characterized by poor economic conditions, a high prevalence of open defecation, and an indigenous belief, called “Marapu”, which is commonly practiced by the locals [11, 12]. In 2018, the level of dropout from school at the ages of 7 to 24 years was 29%, and the ratio of poor people in East Sumba was also high (30%). Moreover, people regularly face severe droughts, which usually occur from April until October [13].

A previous WASH study analyzing the household water treatment (HWT) behaviour was conducted in East Sumba in 2018 [5, 14]. That study identified knowledge gaps that needed to be studied in order to better understand WASH-related behaviour in the area, e.g., the role of culture and environmental conditions. Moreover, unsustainable WASH services in that area, e.g., rural water supply or latrine that cannot be used after some time, were a problem in some places indicating the need to study them in more detail.

In this study, which was conducted in 2019, we adopted a qualitative approach to explore the factors that influence WASH conditions (Fig. 1) [10]. We used some of the previous results of the 2018′ study to support our analysis [5, 14]. Furthermore, the findings were summarised and illustrated in a mind map to show how one factor influences others or the WASH condition. This approach can be used to understand the root causes of a phenomenon or problem, i.e., the beginning of an interconnection pathway. We visualized the results in a mind map to show how various factors, under the FIETS aspects, are interconnected, revealing potential root causes of the WASH conditions in the area.

## Methods

### Guiding theoretical framework: FIETS

We followed the FIETS framework to explore aspects that contribute to the current WASH conditions in East Sumba. FIETS was introduced by the Dutch WASH Alliance (DWA) as a tool to evaluate or monitor the sustainability of their WASH programs in developing countries [15]. FIETS has been widely used as a sustainability framework by WASH-related stakeholders [16], including in Indonesia [17, 18], which was the main reason for choosing it for this study. Moreover, the FIETS framework could be applied to all WASH contexts, e.g., water, sanitation, or hygiene, unlike other sustainability frameworks, e.g., UNICEF’s Sustainability Check (which is for sanitation only) and GIFT (which is for water supply only). Furthermore, some of these sustainability frameworks contain either fewer factors that may not explain the overall conditions, e.g.WaterAid’s Sustainability Snapshot and POSAF, or a larger number of factors that are quite complex to be analysed in the field, e.g., The World Bank’s Enabling Environment Assessment and WASH Sustainability Sector Assessment Tool [19]. FIETS consists of five main aspects, namely (1) *Financial*, (2) *Institutional*, (3) *Environmental*, (4) *Technological*, and (5) *Social*.

*Financial* cover aspects related to economics, such as government or local financing, project financing, community contribution, tariffs, and a household’s economic condition. Previous studies have identified finance-related factors that influence the sustainability of WASH such as: sufficient finances of the project [7], operation and maintenance costs [20], and community financial contribution [21–23].

*Institutional* aspects relate to stakeholder performance, such as government effectiveness, regulation, or corruption [24]. Some factors related to the institutional aspects that have been mentioned are: community participation [7, 25, 26], active water boards [27], support from experienced organizations or government [27–31], leadership [32–34], and regulations [35].

*Environmental* aspects relate to the natural environment and resources that support the sustainability of a WASH program. Some of the environmental factors that may relate to WASH are the effect of a natural disaster on WASH facility [27, 36], distance to the water source which could influence WASH behaviour [37], water availability, especially during the dry season [6, 22, 38], and climate change effects on water quality and availability [39–42].

*Technological* aspects relate to hardware or technology used by the target group, such as technical maintenance. Some of the factors that are often mentioned are supply chain of the technology or distance to the main market [22], maintenance processes [43], quality of the technology [25, 29, 44], and technical knowledge and skill set of the technical officers [45].

*Social* aspects focus on appropriate social conditions to sustain a WASH program or behaviour, like psychological factors that influence a household’s behaviour. Many WASH studies have discussed this, especially psychological factors responsible for the WASH behaviour, such as risk perception [46], social norm [47, 48], sense of ownership [49], or local culture [50].

### Study setting

**Fig. 2:**
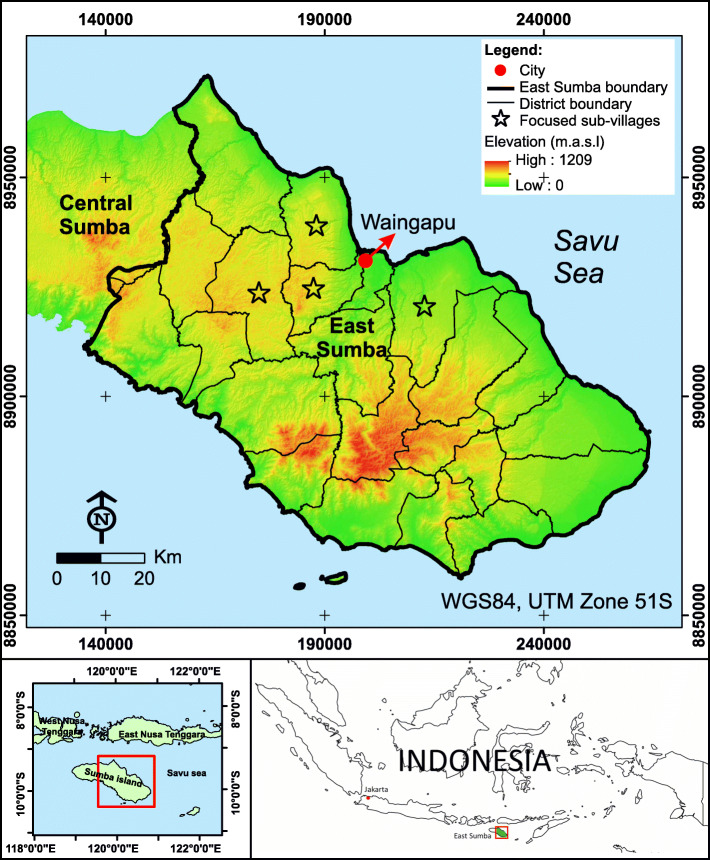
The map of the study area and the focused sub-villages. The map was generated using ArcGIS 10.5 (ESRI, Redlands, CA, USA)

Among all 34 provinces in Indonesia, East Nusa Tenggara had the highest prevalence (42.6%) of child stunting [8]. In 2018, the access to improved drinking water sources in East Nusa Tenggara was 72%, which increased from 63% in 2015, and access to improved sanitation increased sharply from 23% in 2015 to 51% in 2018 [51, 52]. The study took place in one of the districts in East Nusa Tenggara, named East Sumba (Fig. 2). Poor WASH conditions, poor socio-economic conditions, high prevalence of malnutrition, and complex challenges related to WASH were the reason for choosing this district as a study area.

The study was divided into two phases: the first phase was from July – August 2018 and the second one was from July – August 2019. During the first phase, a quantitative analysis of HWT perceptions and adoption was performed. A total of 377 households in four sub-districts in East Sumba were interviewed. More details on this study can be found in [5, 14]. During the second phase of the study, we implemented a qualitative analysis to understand the influencing factors related to WASH. More specifically, we conducted semi-structured in-depth interviews with relevant stakeholders, such as the District Agency for Regional Development, the Health Agency, the Public Works Agency, the Sub-district Board, the Community Based Rural Water Supply and Sanitation Program (PAMSIMAS) officer, the health clinic, and water driller. The respondents were people who were either responsible for or involved in WASH-related projects in that area. At the village level, we interviewed the village head, village council, water board, religious leader, and at least five random households in each of the selected villages. Most of the respondents from households were women, while other stakeholders were dominated by men. Nine villages in four sub-districts were the focus of our research, see [14] for more information about the village selection. In total 91 interviews were conducted and each interview lasted 30–90 min. The questions were structured according to the theme or topic. For example, in the household questionnaire, the themes were water, sanitation, household water treatment, and the stakeholder’s relationship. Question guidelines can be found in Section S4 of the supplementary material. During the time of the interview, there were no NGOs actively executing a WASH program in East Sumba. However, we interviewed five local NGOs that had been active in that area for more than 5 years to gain more insight into the NGOs’ perspectives of WASH conditions. The interview was conducted by three interviewers, i.e., the first three authors, in Bahasa, since the interviewers are native.

### Data analysis

During the in-depth interviews, important points were noted by the three interviewers and discussed together at the end of the day to collate the information. The data collection (interview), analysis, and validation (inter-rater reliability) were conducted by three interviewers. The findings were then categorized into each of the FIETS aspects. We also complemented the analysis with extra information from existing literature, related data from government agencies, Geographic Information System (GIS) modelling, and previous household surveys in 2018 [14]. For example, the issue of a severe drought in East Sumba was strengthened by the results of GIS modelling, using ArcGIS 10.5 (ESRI, Redlands, CA, USA).

We then created a mind map to summarise key factors in all FIETS aspects. We mainly used factors which either were often mentioned by the respondents or factors which were mentioned as critical even though they were only mentioned by one or two respondents. The arrows in the mind map indicate the relationships or interconnections between the factors obtained from the interviews. In this paper, a factor refers to the elements of a WASH system, e.g., local culture, sense of ownership, climate, norms, etc., while an aspect refers to *Financial*, *Institutional*, *Environmental*, *Technological*, and *Social* dimensions The mind map consists of three main levels: (1) level one is the centre - WASH conditions in East Sumba, (2) level two is composed of the five FIETS aspects, and (3) level three is composed of the key factors or elements related to the FIETS aspects derived from the in-depth interviews. The mind map allows us to find the “root causes” of a problem which is indicated by a factor or element located at the beginning of a relationship or an interconnection pathway.

For the factors related to the *social* aspect, we used the RANAS psychological framework [53] to categorize and organize the findings and discussion. This also helps align this research with a previous study in this area [5]. RANAS stands for *Risk* (perceptions regarding health risk related to behavior), *Attitude* (positive or negative views toward a behaviour), *Norms* (social pressure related to behavior), *Ability* (personal confidence to perform the behavior), and *Self-regulation* (self-management or attempts to plan and monitor the behaviour) which are the psychological aspects responsible for people’s behavior. The factors related to the *social* aspect are discussed under these five psychological factors in the mind map. We did not follow a specific framework to organize the findings and discussion in other FIETS aspects besides the *social* aspect.

Lastly, we performed a stakeholder analysis and created an importance-influence matrix to identify key actors and the “victims” of the current situation. “Importance” means the priority given to satisfying stakeholder’s needs and interests, while “influence” means the stakeholders’ power to affect a WASH program [54]. We first made a list of WASH-related stakeholders in that area. To assess a stakeholder’s level of importance and influence, they were assigned a score from 1 (the smallest) to 5 (the biggest) that was used to create the importance-influence matrix. The matrix was divided equally into four quadrants A – D, e.g., quadrant A (top-right left) consists of stakeholders who have an importance score between 2.5–5 and an influence score between 0 and 2.5. In addition, we discussed potential WASH promoters, i.e., agents of promotion, in the study area.

## Results and discussion

All findings were grouped and discussed within the scope of the FIETS aspects. Information related to more than one aspect is placed in the most relevant aspect. The findings are followed by a discussion of the mind map and stakeholder analysis. Finally, we discuss the key challenges and implications of this study.

### Applying the FIETS framework to identify factors related to WASH services

#### Financial aspect

Respondents from district agencies and village boards often mentioned a lack of funds as an obstacle for sustainable WASH services in East Sumba. However, one of our respondents said that there are actually many funding sources for WASH-related activities. For example, a village or appropriate agency can ask for *dana alokasi khusus* (DAK, special allocation fund) from the national government to build or repair WASH infrastructure. Further, we found that the priority of village boards to improve the WASH conditions was often low, as can be observed from the village budget in 2018. In our focused villages, the average village fund allocated for WASH was 2.1% of the total village fund, which we consider is low considering the urgent WASH-related needs for better services in the area, e.g., the average level of open defecation in 2019 was 27.4% [14].

In most villages, subsidies were available from the village offices to construct latrines, i.e. people can obtain materials but they have to construct the latrine themselves, although the materials were given in turns and reached only a few selected poor houses per year. In this case, a sub-village, i.e., the administrative level below the village, gives a list of poor households to the village office and then the village office decides which households receive the subsidies. However, there were beneficiaries who did not construct the latrine, either due to laziness or lack of water for proper use.

Since the final decisions of the beneficiaries were made by the village heads, there were chances of bribery, or arbitrary favouritism by the village heads, e.g., relatives or supporters. For the latter, nepotism was quite often mentioned by our respondents.

The community was expected to contribute financially to WASH services, especially communal tap water. However, there was a tendency that the beneficiaries did not want to contribute anymore after they used the service for some time. The most common reasons mentioned by the beneficiaries were: “the water does not flow anymore”, a suspicion that the water board misused the collected money, or “I saw people from outside the village, who do not pay for the tap, come and take the water from our tap”. In addition, the village water boards said that a low sense of ownership and a lack of understanding of the importance of financial contributions by the community were also the reasons.

It was further found that people in East Sumba tended to spend large amounts of money for cultural events, e.g., funeral and marriage ceremonies. They frequently felt rejected if they did not financially contribute to the cultural event. Therefore, local NGOs argued that people in East Sumba are actually not poor, rather they do not appropriately value the continued provision of WASH services.

#### Institutional aspect

One of the indicators of a strong institution that was mentioned by Hamer et al. [55] is whether there is a district policy regarding the provision of adequate WASH services. At the district level, we found that there were policies and instructions to reduce malnutrition in children and improve the WASH services. These policies require relevant stakeholders to cooperate, e.g., the district health and public work agencies. However, from the interviews with the stakeholders, we found that the communication lines were not well developed and the stakeholders rarely met or communicated. In addition, one of the respondents said that function rotation, i.e., staff movement within an institution, is one of the obstacles for effective collaboration between agencies, since new officers would need some time to reach the level of understanding of the previous officer.

At the village level, we found that many village leaders were not aware of the importance of good WASH practices. Only two villages out of the nine villages that we visited had a village policy regarding the provision of adequate WASH services. These villages also formed a village water board and allocated a budget for WASH services, e.g., for maintaining and repairing the water distribution systems.

All water boards of the villages studied said they were reluctant to penalize households who do not regularly pay for the water service. It was said to be a dilemma because most people in the same village are relatives. Therefore, rule enforcement by the water or village boards was difficult.

We also found the influence of local culture on the institutional aspects. The local belief, *Marapu*, is based on a caste system that affects daily life. A leader in the village or other government agencies has to be from a high caste, i.e., royalty, while people who are from lower castes are considered to be “servants” by the high caste people. This makes the voice of a high caste person more powerful than those of the low caste, making the latter group more vulnerable.

Another output of this social system was the (political) competition between some high caste groups or families. The effect on the provision of WASH services was apparent, particularly after a village or district election. There were some cases in East Sumba, according to our respondents, in which the villages’ aid mainly targeted the supporters or families of the elected leaders.

However, there were also some examples mentioned of good leadership. We found in one village that people who received a subsidy to build a latrine had to sign agreements with the village board in which it was stated that they should finish the construction of their latrine within a predetermined time, otherwise the village would withdraw all the materials. Another example came from the only sub-district in East Sumba that was declared as open defecation free. The sub-district head had been able to persuade all village heads under him to perform *sumpah adat* (custom vow) to eliminate the open defecation. The custom vow was highly appreciated by the indigenous East Sumba people and it worked as an effective stimulant. Moreover, we found some stories of successfully applying the “reward and punishment” approach, especially by the district social agency. For example, in some villages, the “threat” of not receiving social aid by the district social agency was effective to force poor people to construct a latrine.

#### Environmental aspect

From the quantitative interviews [14] it was concluded that most of the households (86%) chose water supply as the most critical and urgent issue in the village to be solved by the municipality or the government. The respondents said that lack of water is the reason for them not to cultivate their land, use the latrine, have a bath every day, or practise handwashing at the five critical times of the day.

The mean annual rainfall of Sumba island is low compared to other areas in Indonesia. The mean annual rainfall in East Sumba is 830 mm/year [13], which is the lowest in East Nusa Tenggara province and far below the mean annual rainfall in Indonesia, i.e., 2702 mm/year [56]. In one of our study areas, the main spring dried out in the dry season and people needed to take water from the neighboring village.

Based on the discussion with the water driller who conducted some studies on the soil structure of the area, it was concluded that the soil structure plays an important role in the water availability in the East Sumba. This argument was supported by a published geological map which shows that limestone dominates the lithology in the North and East part of East Sumba [57]. The map also indicates that groundwater availability is not only controlled by rainfall but also by the lithology below the surface, i.e., the groundwater may be found locally in highly saturated and permeable zones only. The water driller said that the geology of East Sumba influences the costs of groundwater exploration and drilling, since the success rate is highly dependent on the comprehensive results of geological and geophysical surveys to locate the saturated-permeable zones. It was mentioned by some local inhabitants that local geology influences the costs of constructing the latrines as well.

Further, it was mentioned by some interviewees that a national company established a 12,000 ha sugarcane plantation in 2020, and planned to expand it to 50,000 ha in the coming years. Our respondents from NGOs argue that this activity results in extensive water extraction from both rivers and groundwater, as also discussed in another study in that area [58]. In addition, as a result of our interview with a water driller, if the sugarcane plantation is located in a groundwater recharge area then it will surely reduce the groundwater availability. Some NGOs mentioned that it would compromise water availability, especially since the companies started to “monopolize” the usage of the river for their activities.

Due to the limestone aquifers, water hardness was often mentioned by the local households as the main water quality issue, instead of fecal contamination. From the data received from a local, commercial potable water company, the total hardness in their water source, measured by the concentration of CaCO_3_, was about 24 mg/L. Furthermore, almost all respondents whom we interviewed said they use visual inspection to judge the water quality, i.e., high turbidity or precipitation after boiling means that the water quality is poor.

It was further mentioned that scattered settlements is an important reason for the high costs of installing a piped network in East Sumba. The hilly topography (see e.g. Figure 2) and the local culture were mentioned as the reason for the location of settlements. Since tribes in East Sumba often had tribal warfare in the past, they live on hilltops, being able to observe enemy attacks. Moreover, many locals in East Sumba also prefer to stay on their inherited land to avoid ownership disputes.

Despite being “troubled” by the environmental conditions, people in East Sumba still benefit from it. For example, most of the households that we interviewed in the villages mentioned that they did not have to spend money on boiling water because they can obtain the firewood from their own fields.

#### Technological aspect

According to our previous quantitative study, only 51% of the respondents in the surveyed villages practiced HWT regularly. Boiling was the common HWT method that people used (85%) [14]. A commercial water filter, produced in Java island, was also found in several houses in one village. Those were sold by a local, private entrepreneur who received financial support to do so from an external NGO. However, the entrepreneur lost contact with the NGO and lacked the filters to continue the business. In addition, during our household visits, we observed that most of the households used a cloth filter after boiling to remove the precipitates that are formed as a result of water hardness.

Based on the latest report of the East Sumba Bureau of Statistics, only 18% of the households in East Sumba had access to a piped water scheme in 2017, while wells and open sources were used by 44 and 32% of the households respectively [59]. We also found that some households used rainwater harvesting in the rainy season. However, this option has limited impact due to the low frequency and quantity of rainfall in this area (see the section on “Environmental aspects”).

According to the village water boards, the most common reasons for a lack of running water from its piped systems were pipe damages due to contact with animals or flooding in the rainy season, and illegal tapping from the network. Most of the pipes were unprotected and located in open fields, making them vulnerable to livestock that mostly roamed free in and around the villages.

Most of the WASH products were produced outside the island of Sumba. For example, iron pipes were not available and needed to be ordered from Java island. According to one of the district agencies, there had been an initiative to establish sanitation entrepreneur groups by UNICEF in 2015, but they were no longer active due to staff rotation in the related district agencies (see the section on “Institutional aspects”).

#### Social aspect

We used the RANAS framework to organize the findings and the factors related to the psychology of people, i.e., we grouped and ordered the information into: *Risk*, *Attitude*, *Norms*, *Ability*, and *Self-regulation*.

Some of the villagers said that “my grandparents did not die even though they always drink raw water. Why do I need to drink boiled water now?” or “I have drunk raw water during my entire life and there is nothing wrong with me”. These perceptions hindered them from drinking treated water. Another perception related to *risk* was that many people in East Sumba still believed that diarrhoea among children is part of teeth growth and therefore did not take diarrhoea seriously. Furthermore, a common method used by parents to heal children’s diseases is to bath them in corn-boiled water. Despite these facts, the majority of our respondents said that poor water quality is one of the reasons for diarrhoea.

In relation to the *attitude* towards good WASH practices, many people who did not drink treated water said that “I will get headache or flu if I drink boiled water” (beliefs about health disadvantages), or “I am not satisfied if I drink boiled water” (personal feeling), i.e., raw water is colder. For the latter, the hot and dry weather in East Sumba were the reason why locals prefer colder (or fresher) water than boiled or hot water. In addition, some respondents believed that boiled water is *air mati* (dead water) which can cause headaches or flu. However, people who always drink boiled water said the opposite, i.e., raw water causes headaches and flu.

**Fig. 3:**
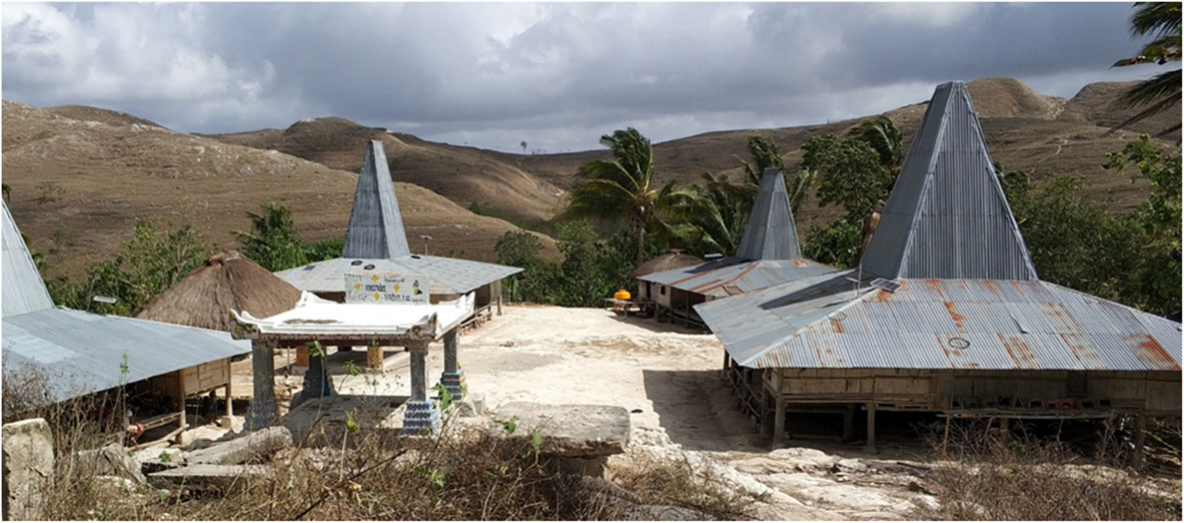
The traditional Sumbanese, stilt, house in one sub-village in East Sumba and the surrounding environment. The picture was taken by the first author

There was no specific social *norm* for HWT practices, but there were norms for sanitation and hygiene practices in the community. Some people who strictly follow a local belief, *Marapu*, did not allow the construction of latrines in their houses or sub-villages due to the perceived impurity of the toilets. A traditional Sumbanese house, i.e., a stilt house (Fig. 3), is a representation of *Marapu* belief, which states that there should be harmony between ancestors, humans, and nature (especially animals). These are represented by the roof, the middle part of the house, and under the house, respectively. The structure of a traditional house does not accommodate a latrine. Therefore, the respondents answered that this had an influence on their practice of open defecation. In addition, respondents from health agencies mentioned that many people in rural areas still believe that faeces need to be given to the animals, especially pigs or chickens, as food. As a consequence, people practice open defecation and the house’s yard is full of dirt.

Another social *norm* among the Sumbanese population, which could inhibit good WASH practices, is a taboo of using the same facility among certain family members. For example, the daughter in law is sometimes prohibited from using the same toilet as her parents in law.

Previous sections have already mentioned some factors that can influence a person’s *ability* to perform certain WASH practices, such as water availability and excessive cultural expenses. Moreover, some respondents said that laziness and a bad mentality among the community members, e.g., hoping to receive some help from outsiders without any intention to improve their WASH conditions, were the main psychological reasons for the poor WASH conditions.

### The FIETS mind map

**Fig. 4:**
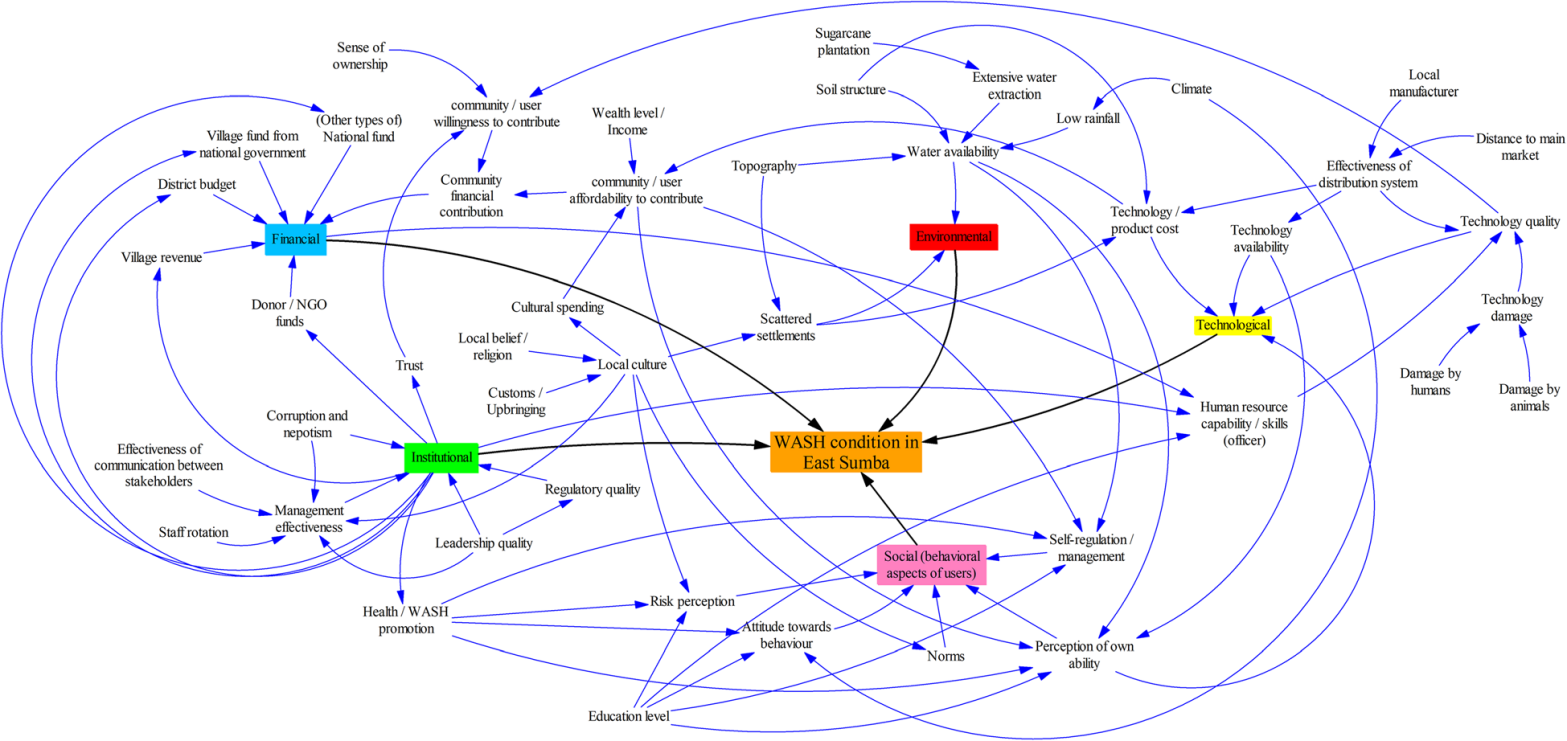
A mind map of factors contributing to the WASH condition in East Sumba. The factors were clustered into five FIETS aspects

All the findings were summarised in the mind map shown in Fig. 4, illustrating the interconnections between the factors responsible for inadequate WASH services in East Sumba. The factors were clustered into the five FIETS aspects. The mind map shows how a factor in one aspect influences the factors in other aspects. For example, as implied previously in the *Financial aspect* section “Financial aspect”, the *institutional aspect* influences *community willingness to pay* via a factor *trust*.

The mind map shows that some of the FIETS aspects had the same “root causes,” i.e., the beginning of a relationship or interconnection pathway, and were related to the socio-economic characteristics (SEC) of people, such as education level, income, distance to the central market, and the local belief. For example, local culture, formed by the local beliefs and customs, indirectly influenced the *institutional*, *financial*, *environmental*, and *social* aspects. The map also shows how “exogenous” factors that cannot be controlled, affected the situation. For example, the climate contributed to the *environmental* and *social* aspects, or the geology influenced the *environmental*, *technological*, and *financial* aspects.

The mind map also exemplifies the interdependencies between the FIETS aspects, for example, the *institutional* aspect influenced the *financial* and *social* aspects, meaning that improving institutional quality will improve the financial and social conditions. Good institutional quality is expected to result in better management of budgets, attracting more donors, and enhancing people’s trust. In this context, the community is then willing to contribute more to a WASH program. Good institutional quality would also result in better WASH implementation and promotional activities which can effectively change people’s behavior. The *Technological* aspect influences the *financial* aspect in the context of a technology’s cost. Moreover, a good institution could provide more training and increase the skills of an implementing officer.

In the cluster of *financial* factors, all possible funding sources for WASH programs were included in the map, including which factors might influence the availability of funding. The willingness to financially contribute to a WASH program is influenced by *trust*, *quality of the technology*, and *sense of ownership*. Moreover, the affordability to contribute is influenced by *cultural spending*, *income*, and *technology cost*.

Furthermore, the mind map summarises four main factors related to the institutional aspects in East Sumba that were often mentioned by interviewees: corruption and nepotism, management effectiveness, leadership, and regulatory quality. Here, we did not distinguish between the scale of stakeholders’ domain in the mind map, e.g., between stakeholders at the village and district level.

There are two main discussion topics related to the *environmental* aspect, according to the interviewees: *water availability* and *scattered settlements*. The difficulty in getting water was due to geology, climate, and extensive water extraction.

The three key discussion topics in the *technological* aspect were costs, availability, and quality. The *technology costs* were influenced by two factors from the *environmental* aspect: *soil structure* and *scattered settlement*, as explained in section “Technological aspect”. The functioning of distribution systems was negatively influenced by the lack of local manufacturers in the area.

The interconnections between factors in the *social* aspect were also inspired by the previous HWT behaviour in East Sumba [14]. The social behaviour of people was thus influenced by their psychological perceptions, such as the perception of *risk* or *attitude* towards good WASH practices. These perceptions were related to their socio-economic characteristics (SEC), such as education or local culture. *Local culture* is a combination of local *Marapu* belief, customs, and individual upbringing.

### Stakeholder analysis

**Fig. 5:**
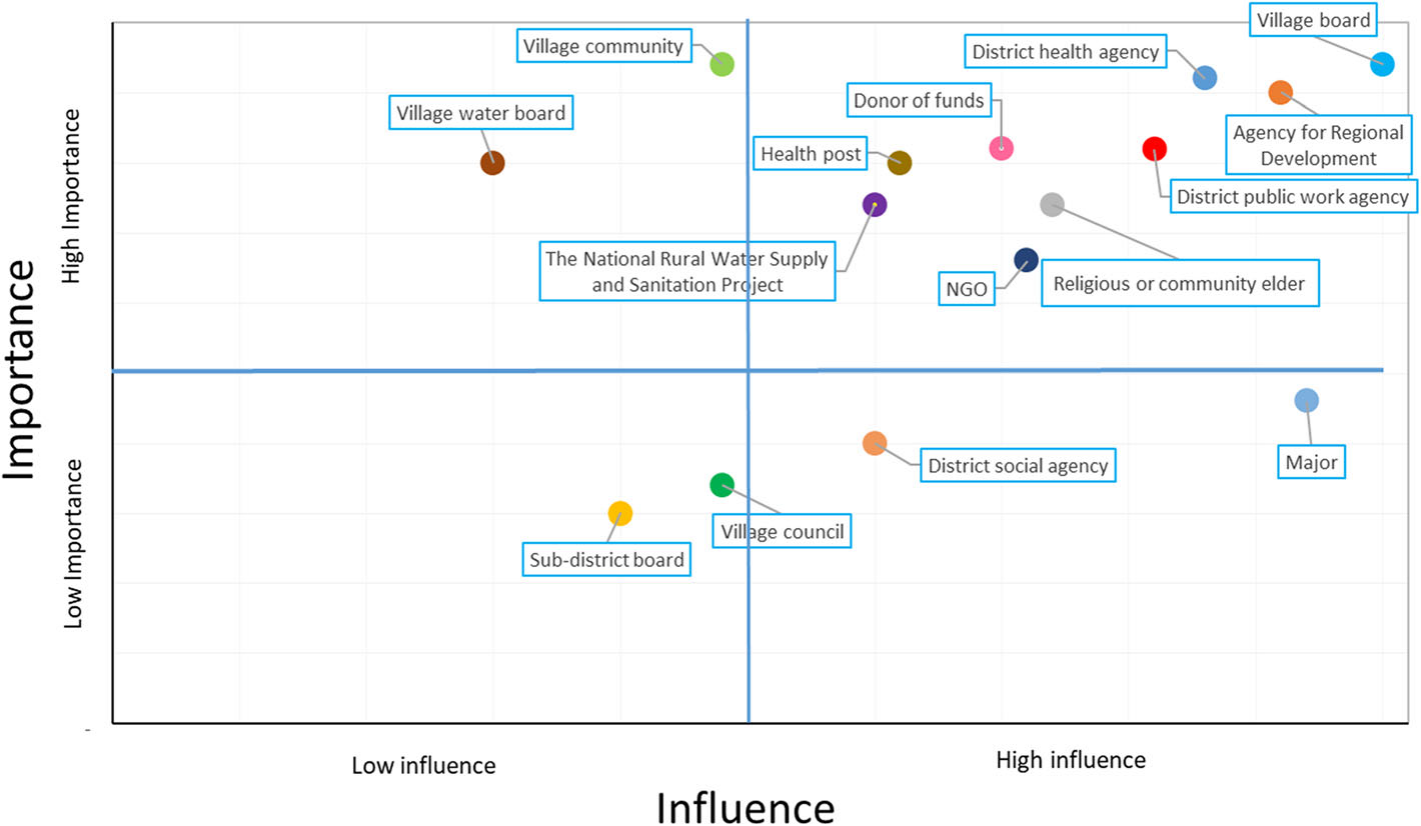
Influence and importance matrix of WASH-related stakeholders in East Sumba

Figure 5 shows the importance-influence matrix of WASH-related stakeholders. Quadrant A shows the groups whose concerns need to be addressed with care, i.e., they are important groups in the project but have a low level of power. The community and the village water boards were in this group. In East Sumba, the influence of village communities on WASH programs was relatively small due to the current social system.

The most important WASH stakeholders in East Sumba are presented in quadrant B, i.e., who can make a difference or decisions, and therefore there must be good working relationships between the stakeholders in this quadrant. A village head or a board has a significant role in improving WASH conditions, followed by the district health agency and the agency for regional development. The influence of a village’s board, especially the village head, was large since most of the village heads were the most respected individuals in the villages and came from higher castes. They also had full authority over the village budgets.

Quadrant C consists of stakeholders who could monitor and evaluate the WASH progress. They should be updated about the program and they could also give recommendations, even though their involvement was relatively low. Sub-district boards and village councils are in this quadrant.

Lastly, stakeholders in quadrant D had limited roles in the program but they needed to be satisfied. Donor agencies, such as the World Bank, the Ministry of economic affairs (national level), or other international funding agencies which give the money via local NGOs, and the district major are in this category. An example of a project supported by those donor agencies is the Community Based Rural Water Supply and Sanitation Program (PAMSIMAS).

In the case of promotional activities, a doctor or health officer was mentioned as the main influential promoter by 62% of the respondents in the study area during the 2018 quantitative study [5]. Als et al. [60], in their review about WASH interventions in developing countries, have mentioned that health officers were rarely reported in previous WASH interventions, whereas they were influential promoters in our study. According to the interviews, WASH promotions were mainly conducted during health consultations, household visits, or antenatal programs. Other promotional activities were at religious places, *Posyandu* (pre and postnatal healthcare information), or schools. Villages in East Sumba usually have more than one *Posyandu*. Promotional activities using social media, radio, or television may not be effective since many houses in rural East Sumba do not have access to electricity. Most of our respondents said that they would start to drink boiled water if they get sick due to suggestions made by their doctors. This was confirmed by the doctors in health clinics who said that they always encourage patients to drink boiled water in every health consultation, especially in case of symptoms which might be caused by unsafe drinking water, such as diarrhoea.

### Key challenges and strategies to improve WASH conditions in east Sumba

Based on the findings, we can argue that East Sumba is vulnerable to unsustainable WASH services. The FIETS analysis reveals three main challenges. First, institutions were not strong enough to support the improvement of WASH conditions. Despite the existence of regulation at the district level, there are weaknesses in its implementation. Second, water scarcity is a problem in this area and WASH services cannot be sustained without proper water provision. Lastly, it is challenging to change people’s behaviour, especially when considering their socio-economic characteristics.

Amongst all the five FIETS aspects, we argue that the institutional aspect is the most critical aspect that influences the WASH condition in East Sumba. The factors in the institutional aspect influence other factors in other four aspects, as recognized widely also in the WASH domain [61–65]. Stronger institutions result in a more reliable financial structure, a higher level of trust by a community, better regulations, better capacity building, and better implementation of adequate WASH services. Moreover, the local culture has a strong influence on the current institutional performance, which may be unique when compared to other WASH studies. The influence of culture on water institutions has begun to attract the attention of water researchers in the past few years, such as in the socio-hydrology domain [66]. However, we found that this is rarely discussed in the WASH sector.

Amongst all the factors that influence the institutional aspect in the mind map, we consider the quality of leadership to be the main cause of institutional problems in East Sumba, which also influences other aspects (Fig. 4). The interview results suggest that the quality of a leader makes a difference in a project’s outcome, not only in the WASH sector, but also in other sectors as well, such as agriculture and economics. Good leadership can positively influence the implementation of, for example, village policies related to WASH or prevent the misuse of a WASH budget. We also argue that the community leaders should be proactive to look for solutions for the WASH-related challenges in their area.

Moreover, an inspirational leader who has much interest in improving WASH conditions was rarely found in East Sumba. Considering the local culture and success stories in East Sumba, finding or nurturing a “champion” or “natural leader”, who can drive the community to sustain or improve WASH services, should be a priority during any WASH implementation. Previous WASH studies have found that this method has been successful in sustaining WASH services in developing countries [29, 67]. Since the rural communities in East Sumba adopt a caste system, the champion should come from a higher caste or should be a respected person, like the community or village leader.

Moreover, the *environmental* aspect in East Sumba cannot be overlooked, especially low environmental capacity to provide water. Previous studies have mentioned that difficult access to water hinders good WASH practices [5, 6, 37], that will probably be worsened by climate change [40]. Extensive groundwater extraction is another factor affecting the water supply in the area and has led to conflicts between the industry sector and native inhabitants [68]. Conflicts to get access to water supply have happened in many parts of the world [69, 70]. Therefore, there is a need to create a district policy regarding groundwater extraction to prevent unsustainable groundwater depletion in the future, especially the extraction by extensive commercial plantations, such as sugar cane that is one of the top four most water-intensive crops [71]. The local municipality should also mediate this conflict by accommodating the interests of all parties and induce cooperation.

Moreover, the Community Based Rural Water Supply and Sanitation Program (PAMSIMAS), which is led by the national government, can be a solution to provide sufficient water to vulnerable communities [72]. However, various initiatives within the program, such as the proposal mechanism, comes from the village office and is submitted to the PAMSIMAS district facilitator, highlighting the importance of local institutions in identifying WASH problems in their areas and seeking potential solutions. The findings related to all FIETS aspects of water supply services should be considered when conducting the PAMSIMAS project, e.g., the potential misuse of budget, dispersed houses, or water theft by people outside the actual beneficiaries.

In addition, the geology influences not only the water services but also the construction of latrines. This situation has also been found in a sanitation program in rural Zambia [73]. That study highlights the importance of design adaption to overcome physical barriers. However, we did not find any specific innovation in latrine designs or constructions in East Sumba.

The case of latrines also shows that there is an overlap of some FIETS aspects. Environmental conditions affect the construction of the water supply systems and latrines, which may trigger the adaptation of the construction (the technical or technological aspects of FIETS) and also influence the capital and maintenance costs (financial aspect). This suggests that WASH topics or the adoption of WASH-related technologies should involve people from different backgrounds in the design and implementation phases. The hope is that the WASH program can be sustained over a long period.

Another important discussion with the NGOs was about an open defecation program led by the national government. The program pushes the local municipality to focus on constructing latrines and reducing open defecation rather than water provision, as also suggested by Firmana et al. [74]. However, we found that inadequate water provision is one of the main reasons why people in East Sumba do not use latrines, even though they have constructed them. In other words, without adequate water provision at home, practicing proper WASH behaviour is difficult [75]. Therefore, we emphasize the need for adding more flexibility to the national policy for locations with “special needs” or water scarcity areas, like East Sumba, to prompt the district governments to balance the focus on water provision and sanitation.

Changing people’s perceptions regarding appropriate WASH behaviour is challenging. Our previous HWT adoption analysis showed that there are some SECs, besides local culture, that influence people’s psychology related to good WASH practices, e.g., low education level, poor wealth status, and difficult access to water [14]. This finding suggests that sustained provision of adequate WASH services depends on a household’s living standard. Thus, the effort to sustain better WASH conditions needs to be accompanied by increasing the living standards of households. This can be a challenge in the case of East Sumba since the district was among the worst 5% in Human Development Index in Indonesia [76]. Our findings also show that local perceptions are not in agreement with the scientific knowledge of water quality [77], since people consider water hardness as a problem even though the value is below the standard. Presenting water hardness, microbial water quality, and their relation to health could remove some of the misconceptions of the target group.

The current WASH conditions cannot be separated from the local cultures as found in other cultural settings [46, 50, 78]. The traditional *Marapu* belief system applies a caste system that determines the social status of people. However, there are several consequences of its practice, since people from a low caste are usually poor and poorly educated, they have limited access to resources, and limited decision-making power [79], leading to inequalities in access to adequate WASH services.

### FIETS framework reveals the factors influencing WASH conditions

This study is an example of the applicability of the FIETS sustainability framework to reveal the factors that influence WASH conditions in a specific area. These factors can be organised into five FIETS aspects and a mind map can be created to visualise how the factors are related. The mind map shows how one factor is related to some other factors in various FIETS aspects. Previous studies have also indicated the potential for interactions to occur between FIETS aspects [65, 80], which support the idea that WASH is a complex issue. The mind map also indicates that, by dealing with the root causes, overall WASH conditions can be improved. For example, improving the leadership quality can improve, either directly or indirectly, the institutional, financial, and social aspects, as shown by the arrows in the mind map. Another example is the influence of local culture. The mind map shows that local culture touches many aspects, e.g., the institutional, financial, environmental, and social aspects. Moreover, the stakeholder analysis revealed important stakeholders and potential WASH promoters, which supports the analysis of the institutional aspect. Therefore, considering the advantages, as described in the methods section, the use of the FIETS framework to analyse the WASH conditions in developing countries is suggested.

## Conclusion

Our study explored the *financial*, *institutional, environmental, technological*, and *social* aspects that contribute to the current WASH conditions in the rural area of East Sumba, Indonesia. The FIETS framework, which is complemented by a mind map and a stakeholder analysis, can unravel the complexity of WASH conditions in developing countries. We explored East Sumba as an example to illustrate the challenges of achieving the sustainable provision of adequate WASH services in the context of vulnerable, indigenous, and rural area of a developing country. We found three main challenges that make East Sumba vulnerable to poor WASH conditions: weak institutions, limited water availability, and poor socio-economic conditions. Additionally, by summarising the key factors obtained from the interviews in a mind map, we discovered the root causes of the current situation and analysed the interconnections between the various factors. For example, local culture is one of the root causes that indirectly influenced all the FIETS aspects. Furthermore, we conclude from the stakeholder analysis that village leaders are key actors who can drive and improve WASH conditions in East Sumba and healthcare workers are important promoters of WASH best practices. We also highlight the importance of analysing the influence of deeply-rooted culture on sustaining adequate WASH services and behavior in developing countries. Finally, we argue that integrating WASH interventions into the prevailing cultural practices is necessary. For example, in our study area, it is important to find a leader from a high caste to trigger the community to adopt water-related technologies in order to improve WASH conditions.

## Supplementary Information

**Additional file 1: S1.** Rainfall per decade in East Sumba. **S2.** Land use map in East Sumba. **S3.** Relationship analysis between socio-economic characteristics and RANAS psychosocial factors. **S4.** Question guidelines for semi-structure interview.

## Data Availability

Data are not publicly available because of the confidentially reason but are available by contacting the corresponding author for reasonable request.
