# Supplementary material for: Financial, institutional, environmental, technical, and social (FIETS) aspects of water, sanitation, and hygiene conditions in indigenous - rural Indonesia
Source: BMC Public Health. 2021 Sep 22;21:1723. doi: 10.1186/s12889-021-11800-x (PMC8459497; doi:10.1186/s12889-021-11800-x)
Supplement: Supplementary file 1 — Additional file 1: S1. Rainfall per decade in East Sumba. S2. Land use map in East Sumba. S3. Relationship analysis between socio-economic characteristics and RANAS psychosocial factors. S4. Question guidelines for semi-structure interview. [file 12889_2021_11800_MOESM1_ESM.docx]

Additional file for

**Financial, Institutional, Environmental, Technical, and Social (FIETS) aspects of water, sanitation, and hygiene conditions in indigenous - rural Indonesia**

D. Daniel^1,2^, Dennis Djohan^1^, Ilias Machairas^1^, Saket Pande^1^, Arifin Arifin^3^, Trimo Pamudji Al Djono^4^, and Luuk Rietveld^1^

^1^Delft University of Technology, Department of Water Management, Faculty of Civil Engineering and Geosciences, the Netherlands

^2^Department of Health Behavior, Environment, and Social Medicine, Faculty of Medicine, Public Health and Nursing, Universitas Gadjah Mada, , Yogyakarta 55281, Indonesia

^3^Bandung Institute of Technology, Department of Groundwater Engineering, Faculty of Earth Sciences and Technology, Indonesia

^4^Sekolah Tinggi Teknologi Sapta Taruna, Indonesia

Corresponding author: D. Daniel ([d.daniel@tudelft.nl](mailto:d.daniel@tudelft.nl))

**This PDF file includes:**

S1. Rainfall per decade in East Sumba

S2. Land use map in East Sumba

S3. Relationship analysis between socio-economic characteristics and RANAS psychosocial factors

S4. Question guidelines for semi-structure interviews

**S1. Rainfall per decade in East Sumba**


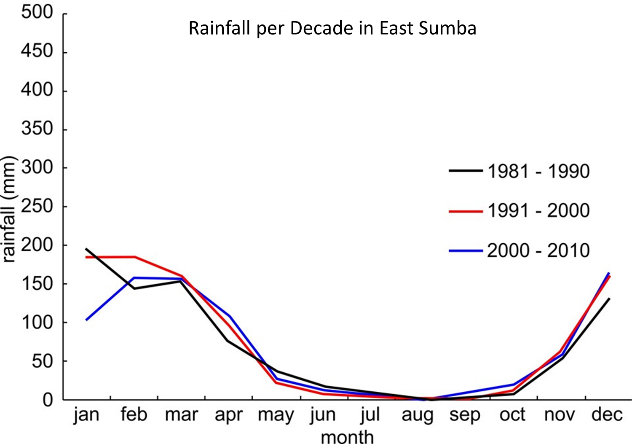


Figure S1. Rainfall pattern from 1981 – 2010. Edited from [1].

From the figure above, the rainfall pattern of the three-decade periods is almost the same. The difference is in the rainfall of January to February. According to the classification used by the Meteorological, Climatological, and Geophysical Agency of Indonesia (BMKG) [2], the rainfall lower than 100 mm/month is categorized as low, while the rainfall between 100 to 300 mm/month is categorized as moderate. Thus, the rainfall in the East Sumba can be classified as low to moderate. In a year, the rainy season in East Sumba occurs from November to April. The previous study found that mean annual rainfall in East Sumba was 830 mm [3], which was the lowest in ENT province and far below the mean annual rainfall in Indonesia, which is 2702 mm [4].

**S2. Land use map in East Sumba**


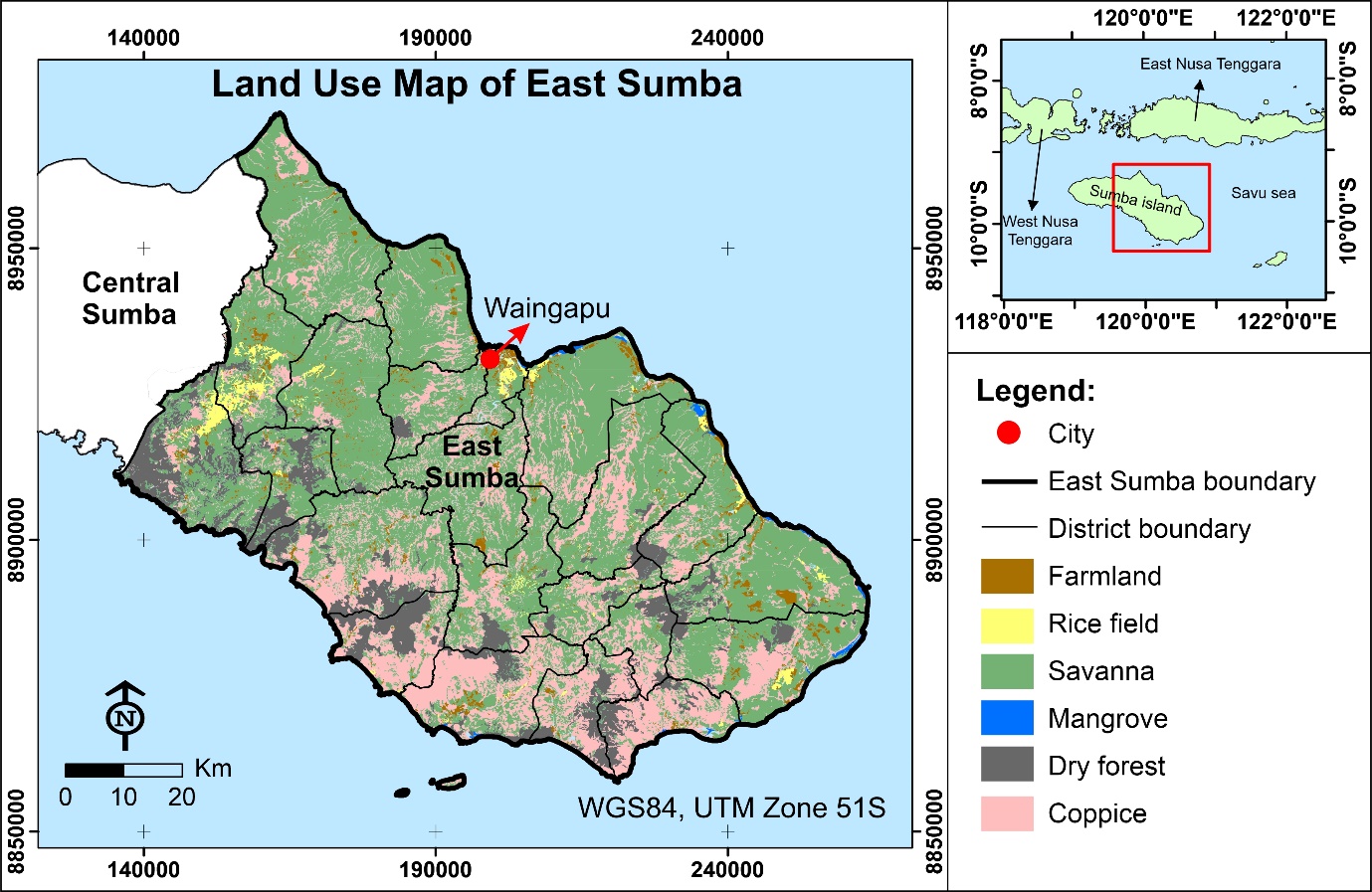


Figure S2. Land use map of East Sumba. This figure was generated using ArcGIS 10.5 (ESRI, Redlands, CA, USA).

From the figure above, savanna dominates the land use of Sumba East Sumba (58.2%), followed by coppice (26.1 %), dry forest (10.7 %), farmland (3.1%), and the other (1.9 %).

**S3. Relationship analysis between socio-economic characteristics and RANAS psychosocial factors**

Figure S6 showed the statistical relationship between selected socio-economic characteristics and 5 RANAS factors in the context of household water treatment practice. This analysis was conducted based on the first phase of the data collection in 2018, i.e., quantitative analysis with 377 households. Please see [5] for more information on the quantitative study.


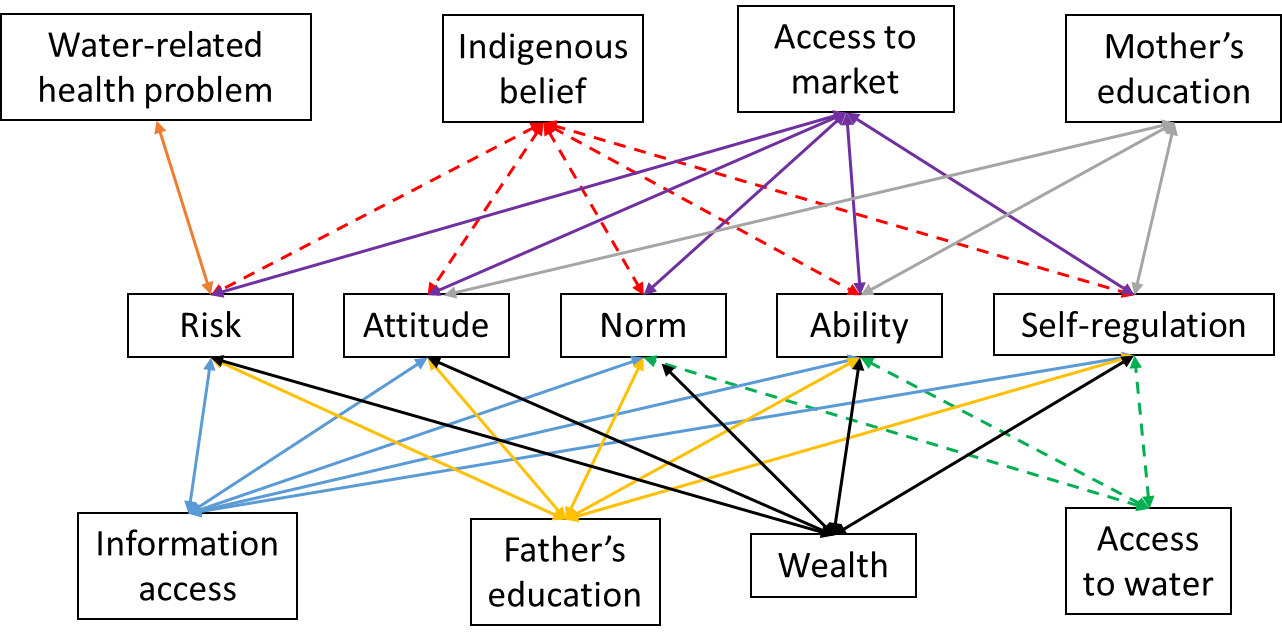


Figure S3. Correlation relationship between socio-economic characteristics and RANAS psychosocial factors. Solid lines indicate positive correlation and dashed lines indicate negative correlation (Pearson correlation, *p* ≤ 0.05).

**S4. Question guidelines for semi-structure interview**

Location (village/sub-village):

Respondent’s Name:

Household’s head name:

Gender:

Age:

Occupation:

Religion:

Highest education:

No. of children:

Diarrhoea problem in the past two weeks:

A. **General Questions for household**

- If you have children, do your children go to school? How many of your children have graduated from secondary school?
- How many goats/cows do you have?
- How many fields/land do you have and what kind of crops?

**Opening questions for the discussion with the household (open question)
*Water Source***

What is your main drinking water source?

Is it private or public (public well, private well, river)?

Do you take the water from somebody else (water company, municipality)?

For which uses do you consume domestic water and in what percentage?

How much do you pay?

Is your water resource reliable?

How do you judge the quality of your water source and/or your drinking water?

If the water deteriorates, what do you do?

Who in your house decides on where to get the water supply?

Who is responsible to bring drinking water to the household?

Who do you think can influence and make a change in the drinking water quality in your region ?

***Water Treatment***

Are you satisfied with the current water quality?

Who do you think in your house is in charge or responsible for the water quality that you have?

Do you treat the water?

If yes, how?

Is your method reliable?

Are there any problems related the method which you use?

If not:

Why don’t you treat your water?

Can you think of any factor, such as culture, religion, that influence your decision not to treat the water?

Is it expensive to treat the water?

How much money are you willing to pay for cleaning drinking water?

Do you boil your water?

If yes:

What is your opinion of boiled drinking water?

Do you like the taste of boiled water?

Is it difficult to get all resources which are needed to boil your drinking water?

How familiar are you with these alternative drinking water procurement (1-10).

Explain briefly the methods (effectiveness and costs relative to each other) and ask for their preferences for each (1-10

Boiling water

SODIS

Membrane filter

Direct pipe network to house

Pipe network to centralized location

Truck deliveries

Bottled water (gallon)

***Sanitation-WASH***

Are you satisfied with your hygiene situation?

What kind of challenges do you face to do proper WASH behaviour?

Who is the person (stakeholder) that can influence your WASH or drinking water habit?

How often do you wash your hands?

Have you ever got sick from the bad water quality ?

Are doctors or NGOs active to help in this case?

Are you satisfied with the doctors services?

Do you take advice about what to drink or not by the doctors?

How important is the doctor’s opinion for you on influencing your WASH behaviour?

***Stakeholder Relationships***

Who is helping you related to water problems (doctors, municipality, water company, NGO)?

Do you cooperate with other stakeholders?

Are you satisfied with the water service from the municipality?

Are you satisfied with the water service from the water company?

Do you know any NGO (Non Governmental Organisation)?

Do you think that NGO and health (agency) collaborate or they act independently?

Do you think that there is a smooth cooperation between local authorities (mayor) and NGOs?

Are you satisfied with the services of water utility company and the municipality according to the water quality and quantity?

Do you believe that all related stakeholders have similar goals or conflicting interests?

Who do think is the most important stakeholder related to water resources/water management in this region?

If there are any problems with water, is there anything that could be improved?

Do you think universities can play a role to this?

Can you think some possible cooperation among stakeholders in the future in order to enhance the water quality?

What changes would you like to apply in your region regarding water sanitation and drinking water?

**B. Open question for the discussion with the water supplier**

***General***

What is the current status of people without proper water, sanitation, and hygiene (WASH) facilities in this area?

Are you a public or private company?

Did you apply or build any structure to any of the villages (Mondu, Hamba Prang, Kuta, Pambotanjara, Mbatakapidu, Makamenggit, Pulu Panjang, Kawangu, Palakahembi) to provide tap water?

Do you check the water quality you deliver?

Are there any standards that you need to meet?

How do you ensure the quality of water? Do you apply any kind of water treatment before delivering the water to the consumer?

Do you have any problems with distribution or treatment?

To whom do you distribute the water?

How many people are connected?

How many people do they have safe water out of the total population of the region?

Do you have plans to extend the network?

Do you give information to the locals on basic hygiene and water management?

Have you ever received acute criticism from a local opposing your plans for better quality drinking water?

***Cost***

What about the total budget (expenditures). Which fraction is going to water resources?

How much money do you allocate for water treatment, water delivery and maintenance of the network?

What is the main source of revenue for the company in order to treat or deliver the water (taxes, regency of East Tenggara)?

***Stakeholder Relationships***

Do you cooperate with other stakeholders? If yes, which ones? Do you cooperate with other stakeholders? If yes, which ones?

Do you exchange technical knowledge with other water utilities in East Tenggara Regency or in Indonesia in general?

Is it required to take approval for every action from local authorities or do you act independently?

Do you believe that all related stakeholders have similar goals or conflicting interests?

Who do think is the most important stakeholder related to water resources/water management in this region?

Can you think of some possible cooperation among stakeholders in the future in order to enhance the water quality?

What changes would you like to apply in your region regarding water sanitation and drinking water?

**C. Open question for the discussion with Pamsimas (National Rural Water Supply Project)**

What are your responsibilities?

What is the annual budget and how you distribute it? Where do you get the income?

How do people judge the quality of their water source and/or drinking water?

Can Pamsimas influence considerably the opinion of locals?

**WASH**

Is there any policy from municipality regarding WASH? If yes, when was it launched? What is the policy? How is the progress?

Do you have an example of village or community in East Sumba which is successful to adopt a new or better WASH behaviour? What is the reason for that?

Do you have an example of village or community in East Sumba which is unsuccessful to adopt a new or better WASH behaviour? What is the reason for that?

Is there any specific belief [Marapu] regarding WASH and diarrhea among people / community in East Sumba? Does it support a good WASH behaviour or not?

Do you experience any conflict of interest in any WASH program (someone or group of people who try to hinder or steer the WASH project)? Can you give an example? Is it fixed now? If so, how did you solve the conflict?

***Stakeholder Relationships***

Do you cooperate with other stakeholders? If yes, which ones? How many times do you meet in average per year or per month?

Is it required to take approval for every action from local authorities or do you act independently?

Do you believe that all related stakeholders have similar goals or conflicting interests?

Who do think is the most important stakeholder related to water resources/water management in this region?

Can you think of some possible cooperation among stakeholders in the future in order to enhance the water quality?

What changes would you like to apply in your region regarding water sanitation and drinking water?

**D. Open question for the discussion with Bupati (District-mayor)**

What is the current status of people without proper water, sanitation, and hygiene (WASH) facilities in this area?

Which are your responsibilities regarding water distribution?

Do you have any plans to improve the current situation?

Did you build any structure to any of the villages (Mondu, Hamba Prang, Kuta, Pambotanjara, Mbatakapidu, Makamenggit, Pulu Panjang, Kawangu, Palakahembi) to provide tap water?

Are the villages homogeneous (inhabitants, water resources, wealth, education)? Do you apply the same policies and decision making in all the villages?

***WASH***

Is there any policy from he province regarding WASH? If yes, when was it launched? What is the policy? How is the progress?

Do you have an example of village or community in East Sumba which is successful to adopt a new or better WASH behaviour? What is the reason for that?

Do you have an example of village or community in East Sumba which is unsuccessful to adopt a new or better WASH behaviour? What is the reason for that?

Is there any specific belief regarding WASH and diarrhea among people / community in East Sumba? Does it support a good WASH behaviour or not?

***Stakeholder Relationships***

Do you cooperate with other stakeholders? If yes, which ones? Do you cooperate with other stakeholders? If yes, which ones?

Do you exchange technical knowledge with other provinces in East Tenggara Regency or in Indonesia in general?

Is it required to take approval for every action from superior authorities (Ministry, Regency) or do you act independently?

Do you believe that all related stakeholders have similar goals or conflicting interests?

Who do you think is the most important stakeholder related to water resources/water management in this region?

Can you think of some possible cooperation among stakeholders in the future in order to enhance the water quality?

What changes would you like to apply in your region regarding water sanitation and drinking water?

**E. Open question for the discussion with Bappeda (district development planning agency; tbc.)**

***General***

What is the current status of people without proper water, sanitation, and hygiene (WASH) facilities in this area?

Which are your responsibilities regarding water distribution?

Do you have any plans to improve the current situation?

Did you build any structure to any of the villages (Mondu, Hamba Prang, Kuta, Pambotanjara, Mbatakapidu, Makamenggit, Pulu Panjang, Kawangu, Palakahembi) to provide tap water?

Are the villages homogeneous (inhabitants, water resources, wealth, education)? Do you apply the same policies and decision making in all the villages?

Do you give information to the locals on basic hygiene and water management?

Have you ever received acute criticism from a local opposing your current and future plans?

**WASH**

Do you have any policy or regulation regarding WASH?

If yes, when was it launched? What is the policy? How is the progress?

Is there any special budget for WASH program in East Sumba? If yes, what is the percentage in the yearly district budget (APBD)?

Does it remain the same in the last 5 years?For what kind of project/expenses is the money spend for? Is it effective?

Do you have any target regarding WASH issue in East Sumba?

Does WASH is one of priorities in East Sumba? In which position? Why?

Is there WASH “special unit” (id=pokja) in East Sumba? Who are they? Do they work as expected? What is the reason for that (yes or not going well)? Do they meet regularly? Do you think that they work in synergy, why?

How important is the mayor’s/municipality representatives’ opinion for you on influencing the WASH behaviour of locals?

What kind of challenges do you face in applying that policy or plan?

Do you think that local religion/culture influences significantly the locals behaviour regarding water?

Are they willing to change their current attitude and mentality or are they reluctant. If so, why?

***Cost***

Do you receive any taxes from locals regarding water management issues? If yes, how much is it per capita?

What about the total budget (expenditures). Which fraction is going to water resources?

What is the main source of revenue for the municipality regarding water issues?

Do you think that you have enough money for the water resources management?

***Stakeholder Relationships***

Do you cooperate with other stakeholders? If yes, which ones? Do you cooperate with other stakeholders? If yes, which ones?

Do you exchange technical knowledge with other provinces in East Tenggara Regency or in Indonesia in general?

Is it required to take approval for every action from superior authorities (Ministry, Regency) or do you act independently?

Do you believe that all related stakeholders have similar goals or conflicting interests?

Who do think is the most important stakeholder related to water resources/water management in this region?

Can you think of some possible cooperation among stakeholders in the future in order to enhance the water quality?

What changes would you like to apply in your region regarding water sanitation and drinking water?

**F. Open question for the discussion with the local health agency / doctor**

***General***

What is the current status of people without proper water, sanitation, and hygiene (WASH) facilities in this area?

What are your responsibilities?

What is the annual budget and how you distribute it? Where do you get the income?

Do you focus more on the personeel or on the infrastructure/

What kind of diseases are you dealing with?

Diarrhea rate in East Sumba:

Stunting rate in East Sumba:

How accessible is a doctor for a local?

In which season are the diseases more prominent? And why?

Is there any group which is more vulnerable to diarrhea?

How high is the incident of diarrhea or cholera in this district?

Do you notice any abnomalies in the development of children at a young age?

Do you face any problem regarding the lack of medicine/ equipment for the proper treatment of the patients within the area?

Do locals opt for visiting an official doctor instead of alternative medication (herbs)?

Is it common for a local inhabitant to visit Waingapu for health issues?

Do you think that local religion (Marapu)/culture influences significantly the locals’ behaviour regarding water?

Are the diseases homogenous among the villages or do you think that each village has different prominent disease?

Do they pay for health services? If yes, how much?

Are you satisfied with the health service?

Do the locals change their attitude towards water use after an incident of diarrhea in their family?

**WASH**

Do you promote/inform healthy WASH behaviour to the patients or local people? Is there any health promotion conducted in the village? Is it regular? By who? What is the content?

Do you promote also water, sanitation, and hygiene?

Which one is the priority of WASH program in East Sumba: (1) reducing open defecation, (2) handwashing, (3) household water treatment, (4) domestic solid waste, (5) domestic liquid waste

Do you have any target regarding WASH issue in East Sumba?

Does WASH is one of priorities in East Sumba? In which position? Why?

Is there any special budget for WASH program in East Sumba? If yes, what is the percentage in the yearly district budget (APBD)? Does it remain the same in the last 5 years? For what kind of project/expenses is the money spend for? Is it effective?

Is there WASH “special unit” (id=pokja) in East Sumba? Who are they? Do they work as expected? What is the reason for that (yes or not going well)? Do they meet regularly? Do you think that they work in synergy, why?

Do you think that people have a good understanding regarding WASH issue?

Do you have any example of village or community in East Sumba which is successful to adopt a new or better WASH behaviour? What is the reason for that?

Do you have example of village or community in East Sumba which is unsuccessful to adopt a new or better WASH behaviour? What is the reason for that?

Is there any specific belief regarding WASH and diarrhea among people / community in East Sumba? Does it support a good WASH behaviour or not?

Do you have data about the progress of access to clean water in East Sumba every year?

How do people judge the quality of their water source and/or drinking water?

Do you do sanitary inspection? Is it regular? By who? Who is the target? What do they do? Can we have the data? Do you include water quality aspect?

How many sanitarians are in East Sumba? What do they do? Where are they located? Are they enough to do all the tasks?

Do you experience any conflict of interest in any WASH program (someone or group of people who try to hinder or steer the WASH project? Can you give an example? Is if fixed now? How do you solve the conflict?

Is there any cultural/religion (marapu) issue regarding WASH? Can you mention?

***Stakeholder Relationships***

Do you cooperate with other stakeholders? If yes, which ones? Do you cooperate with other stakeholders? If yes, which ones?

Do you believe that all related stakeholders have similar goals or conflicting interests?

Who do think is the most important stakeholder related to water resources/water management in this region?

Can you think of some possible cooperation among stakeholders in the future in order to enhance the water quality?

What changes would you like to apply in your region regarding water sanitation and drinking water?

**Open question for the discussion with the local NGO**

***General***

What is the current status of people without proper water, sanitation, and hygiene (WASH) facilities in this area?

What are your actions/responsibilities regarding water issues?

Do you provide any health service (medical support, doctors) to the locals regarding health issues?

Did you build any structure to any of the villages (Mondu, Hamba Prang, Kuta, Pambotanjara, Mbatakapidu, Makamenggit, Pulu Panjang, Kawangu, Palakahembi) to provide tap water?

What are the main differences among the above-mentioned villages?

Do you believe that all the villages of interest face similar water problems?

Do you give information to the locals on basic hygiene and water management?

Have you ever applied or planned a similar project related to WASH?

What kind of challenges do you face in your work?

Do you think that local religion/culture influences significantly the locals behaviour regarding water?

Are locals open-minded and do they want to change their mindset towards better water quality?

Have you ever received acute criticism from a local opposing your current and future plans?

Which kind of families/individuals accept easily your suggestions?

***Cost***

What about the total budget (expenditures). Which fraction is going to water resources?

What is the main source of revenue for the NGO?

Do you think that you have enough money for the water resources management?

***Stakeholder Relationships***

Do you cooperate with other stakeholders? If yes, with which ones?

Do you believe that all related stakeholders have similar goals or conflicting interests?

Who do think is the most important stakeholder related to water resources/water management in this region?

Can you think some possible cooperation among stakeholders in the future in order to enhance the water quality?

What changes would you like to apply in your region regarding water sanitation and drinking water?

How important is the municipality’s opinion for you on influencing your actions?

What communication difficulties do you face in collaboration with other stakeholders?

**G. Open question for the discussion with the Water Kiosks**

Name:

Address:

What is the price for water and what is the quanity which they usually buy it?

How often do people come to your store so as to take water?

From which villages do they come from? Could you quantify their percentages approximately?

What is the economic status of the people who come to the store so as to purchase water?

***Stakeholder Relationships***

Do you cooperate with other stakeholders? If yes, with which ones?

Do you believe that all related stakeholders have similar goals or conflicting interests?

Who do think is the most important stakeholder related to water resources/water management in this region?

Can you think some possible cooperation among stakeholders in the future in order to enhance the water quality?

What changes would you like to apply in your region regarding water sanitation and drinking water?

How important is the municipality’s opinion for you on influencing your actions?

What communication difficulties do you face in collaboration with other stakeholders?

**References**

1. BMKG. Informasi Perubahan Normal Curah Hujan. https://www.bmkg.go.id/iklim/perubahan-normal-curah-hujan.bmkg. Accessed 4 Nov 2019.

2. BMKG. Analisis Curah Hujan dan Sifat Hujan Bulan September 2019. https://www.bmkg.go.id/iklim/informasi-hujan-bulanan.bmkg?p=analisis-curah-hujan-dan-sifat-hujan-bulan-september-2019&tag=&lang=ID. Accessed 4 Nov 2019.

3. Messakh JJ, Moy DL, Mojo D, Maliti Y. The linkage between household water consumption and rainfall in the semi-arid region of East Nusa Tenggara, Indonesia. IOP Conf Ser Earth Environ Sci. 2018;106.

4. The World Bank. Average precipitation in depth (mm per year) - Indonesia. The World Bank. 2014. (Messakh, Moy, Mojo, & Maliti, 2018). Accessed 4 Nov 2019.

5. Daniel D, Pande S, Rietveld L. The effect of socio-economic characteristics on the use of household water treatment via psychosocial factors: a mediation analysis. Hydrol Sci J. 2020;65:2350–8.
